# Early predictors of reading success in first grade

**DOI:** 10.3389/fpsyg.2023.1140823

**Published:** 2023-08-14

**Authors:** Ana Sucena, Cristina Garrido, Cátia Marques, Marisa Lousada

**Affiliations:** ^1^School of Health, Polytechnic Institute of Porto, Porto, Portugal; ^2^CINTESIS.UA@RISE, School of Health Sciences (ESSUA), University of Aveiro, Aveiro, Portugal

**Keywords:** reading, predictors, letter-sound knowledge, mother’s education, intermediate orthography

## Abstract

Reading acquisition is a complex process that can be predicted by several components which, in turn, can be affected by the orthography depth. This study aims to explore the early predictors of (un)success in reading acquisition within an intermediate transparent orthography. At the beginning of the school year, 119 European Portuguese-speaking first graders were assessed regarding (i) sociodemographic variables: mothers’ education and socioeconomic status (SES); (ii) cognitive variables: phonological working memory and vocabulary; (iii) reading-related variables: letter-sound knowledge, phonemic awareness, and rapid naming. Results of the three variable clusters were correlated with the final classification obtained in the Portuguese discipline. Specifically, there was a correlation between the Portuguese discipline classification with all reading and cognitive-related variables, with the highest correlations occurring with mother education and letter spelling. A regression analysis was conducted to assess the predictor impact of mother education and letter spelling (variables that correlated stronger with the Portuguese classification) on Portuguese classifications. Letter spelling was the sole significant predictor of the Portuguese classification. Based on these results, a path analysis was run to test whether letter spelling is a mediator of the relationship between the mother’s education and the Portuguese classification. The results of the model test yielded a reasonable fit, indicating a relationship between the mother’s education and letter spelling, which in turn, relates to the Portuguese classification. The identification of reading predictors in an intermediate-depth orthography such as European Portuguese contributes to more accurate identification of at-risk children.

## 1. Introduction

Reading acquisition constitutes a fundamental initial stage within the educational context, serving as a pivotal means to acquire the cognitive reservoirs essential for academic proficiency and subsequent occupational prospects (Jamshidifarsani et al., 2019). Reading difficulties in childhood are known to be a risk factor: students with reading difficulties are more likely than their peers to school dropout or choose vocational education (e.g., Hakkarainen et al., 2015). In this way, promoting reading acquisition is a primary goal of the basic education system (Direção Geral da Educação [DGE], 2018). Despite this goal, in Portugal, the academic retention average in 2nd grade is around 9% in the last 10 years (PORDATA, 2021). The academic retention measure is the most serious measure of compensation for learning disabilities. However, grade retention can have a negative effect in the areas of growth, learning, social adjustment, and classroom behavior (Reutzel et al., 2005; Jimerson and Ferguson, 2007; Griffith et al., 2010; Reynolds et al., 2010; Dombek and Connor, 2012). Early and accurate identification of at-risk children with reading disabilities is essential to allow targeted and early intervention that may prevent reading difficulties or reduce their impact (e.g., Fien et al., 2015). Practitioners would be in a better position to early identify and allocate resources to at-risk children if more accurate information about the reading predictors is available.

Among the main predictors for success in reading acquisition, research indicates three clusters of variables: sociodemographic, cognitive, and reading-related. Generally, different studies have shown that sociodemographic variables such as SES (Corso et al., 2016; Freitas et al., 2019) and mother education (Direção-Geral de Estatísticas da Educação e Ciência [DGEEC], 2016; Guo et al., 2018) affect reading skills. The SES is a composed measure that correlates with unsuccessful learning paths, specifically in reading. This result has been explained by the high prevalence of undereducated people in low-SES communities. Also, inadequate education and increased dropout rates initially affect children’s reading skills with an impact on their later academic achievement, perpetuating the community’s low SES (Aikens and Barbarin, 2008). Other studies (e.g., Archibald et al., 2019) explain the relationship between cognitive and reading measures based on a cause-effect relationship, i.e., better cognitive skills, such as vocabulary and working memory, would be the basis for better reading competence. Reading involves understanding the meaning of words, and a strong vocabulary is necessary for successful reading comprehension. A person’s ability to recognize and understand words is largely determined by their vocabulary knowledge (Ouellette, 2006).

Among the sociodemographic predictors, the role of the mother’s education has been indicated as the most closely linked variable to school abilities at an early stage of schooling in transparent orthographies (Finish – Koponen et al., 2016) and semi-transparent (Polish – Zalewska-Lunkiewicz et al., 2016). A strong correlation with educational achievement, specifically reading, is a common result found in families from disadvantaged socioeconomic backgrounds among Chinese children (Guo et al., 2018). On the other end, children whose mothers have a higher level of education are at an advantage. There is a very strong correlation between mothers’ education and children’s educational success. Those children whose mothers have a bachelor’s degree tend to have a significantly higher success rate than those whose mothers did not go beyond the 4th grade (Direção-Geral de Estatísticas da Educação e Ciência [DGEEC], 2016). A higher mother’s education is considered a protective factor for successful reading acquisition (Elbro et al., 1998; Direção-Geral de Estatísticas da Educação e Ciência [DGEEC], 2016; Koponen et al., 2016).

Among the cognitive predictors, vocabulary has been associated with reading skills and specifically with reading difficulties in different orthographies (Volkmer et al., 2019). Also, regarding cognitive predictors, another variable commonly found in the literature is phonological working memory (Teng and Zhang, 2021). Phonological working memory can be assessed through nonword or digit tasks. Nonword tasks have been commonly used in studies of bilingualism (e.g., Chiat and Polišenská, 2016). Digit memory tasks have been implemented in predictive and association studies between reading variables (e.g., Ziegler et al., 2010). However, phonological working memory was a weak and highly inconsistent predictor of literacy development in different orthographies (Caravolas et al., 2012). Earlier studies indicate that phonological working memory problems are related to reading difficulties and are a mediator of the relationship between Rapid Automatized Naming (RAN) and decoding (Papadopoulos et al., 2016; Cowan et al., 2017). Phonological working memory is a reading predictor, especially during the beginning years of schooling (Schaars and Verhoeven, 2019). A discrepancy between intelligence quotient (IQ) and achievement is often considered to explain learning disabilities (Eloranta et al., 2017). However, the association between IQ and reading difficulties is unclear (Eloranta et al., 2017; Cornoldi et al., 2019). Although intelligence is considered independent from the development of reading difficulties *per se* (e.g., American Psychiatric Association, 2013; Eloranta et al., 2017), reading comprehension problems are especially related to components of verbal intelligence and vocabulary in particular (e.g., Swanson et al., 2018). Specifically, Peng et al. (2018) found nonverbal IQ to be a predictor of reading comprehension. Several studies report a positive correlation between vocabulary extension and reading acquisition, especially in transparent orthographies such as German (Volkmer et al., 2019), suggesting that vocabulary influences reading comprehension (e.g., Ouellette, 2006; Collazos-Campo et al., 2020; Cadime et al., 2021). These findings highlight the importance of improving oral skills before early reading acquisition.

Among the reading predictors, letter-sound knowledge, phonemic awareness, and RAN have been the most extensively studied (e.g., Eloranta et al., 2017; Gordon et al., 2020) in opaque and transparent orthographies (Caravolas et al., 2013; Landerl et al., 2022). These predictors are considered key pre-reading skills for reading acquisition (Kyle et al., 2013; Viana and Sucena, 2014; Sucena et al., 2015). Individual differences in letter-sound knowledge are among the most robust early predictors of reading skills (Guaraldo et al., 2020). The letter-sound knowledge - letter reading and letter spelling - represents the foundation for decoding and spelling. The child’s knowledge of letter names reflects the child’s ability to associate phonological codes with graphic symbols (Carvalho et al., 2017). Letter-sound knowledge predicts success in reading acquisition right from the beginning of first grade and even in preschool in those cases when letter-sound is formally taught (Vaessen et al., 2010; Salvador and Martins, 2017; Volkmer et al., 2019). In the same way, the performance on spelling and decoding is strongly related to phonemic awareness and letter-sound knowledge (Taha and Saiegh-Haddad, 2015). Phonemic awareness is known to predict reading acquisition at early stages (e.g., Papadopoulos et al., 2016; Volkmer et al., 2019). Phonemic awareness tasks such as segmentation and phonemic manipulation are strong predictors of reading skills (such as letter reading and letter spelling) in first grade (Snowling, 2014; Porta and Ramirez, 2019). Letter-sound knowledge, phonological awareness, and rapid automatized naming were found to be common predictors across opaque and transparent orthographies (Ziegler et al., 2010; Landerl et al., 2022). Furthermore, phonological awareness seems to be an important factor associated with reading performance in several languages with different levels of orthographic transparency and transversal throughout the first 4 years of schooling. However, their association seems to decrease over the school years (Ziegler et al., 2010). Thus, these competencies should be the main focus of early intervention (Foorman et al., 2016). Phonemic awareness is the ability to identify the sounds of words. It is a foundation skill for reading acquisition that facilitates understanding phoneme-grapheme correspondences (Hulme et al., 2012; Snowling, 2014). Phonemic awareness is usually developed in the context of reading acquisition (Landerl et al., 2018), however, it is possible to develop phonemic awareness before reading acquisition (Lundberg et al., 1988; Sucena et al., 2022). Children that start formal education (6 years old in Portugal) without phonemic awareness might struggle to understand letter-sound correspondences (Sucena et al., 2021). RAN is assessed by the speed with which a stimulus is named, assessing the ability to fluently retrieve and name familiar items (Gordon et al., 2020). In RAN, the stimulus can include only one semantic category (e.g., colors, letters, numbers, or objects) or different categories simultaneously (e.g., objects and colors). Tasks with letters and numbers are more discriminative whereas tasks with objects and colors are considered better predictors of reading ability (Lúcio et al., 2017). Slow performance on these tasks is associated with poor reading performance (Stappen and Van Reybroeck, 2018). Specifically, RAN is closely related to reading fluency in childhood (e.g., Papadopoulos et al., 2016) having an impact in later phases (3rd or 4th grade) of reading acquisition (Kuperman et al., 2016; Volkmer et al., 2019), when the reading speed almost doubles and there is a transition from alphabetical to orthographic decoding. The performance of RAN has also been found to predict developmental dyslexia across several orthographies, from the most opaque like English or French to the most transparent like, German, Dutch, and Greek (Landerl et al., 2018).

The moderating role of the above-mentioned skills has been widely documented in opaque orthographies such as English (Rao, 2018) or French (Landerl et al., 2018), but also Slovak and Czech (Caravolas et al., 2013), as well as in transparent orthographies such as Finnish, Spanish, Italian, or Greek in children at the reading acquisition phase (Ferroni et al., 2018; Freitas et al., 2018; Stappen and Van Reybroeck, 2018; Caravolas et al., 2019; Gorgen et al., 2021), but few studies with an intermediate orthography such as European Portuguese (Vaessen et al., 2010; Ziegler et al., 2010; Carvalho et al., 2017) have been conducted. In Ziegler et al. (2010) study, conducted with 2nd graders of five languages with different orthographic transparency (Finnish, Hungarian, Dutch, Portuguese, and French), the variables phonological awareness, memory, vocabulary, rapid naming, and nonverbal intelligence were investigated. The results indicated that phonological awareness was the main factor associated with decoding and reading accuracy, across the five languages. However, with a stronger association in less transparent orthographies. RAN showed weak associations. Memory had an association with accuracy and decoding only in Finnish and Hungarian and nonverbal intelligence had no significant association. In turn, Vaessen et al. (2010) studied reading fluency from 1st to 4th grade in three orthographies with different transparency (Hungarian, Dutch, and Portuguese). Results indicated that phonological awareness kept a significant association across all grades, decreasing as a function of the year. The RAN association increased over the grades. The orthographic transparency of the different languages did not change the strength of the associations. Carvalho et al. (2017) conducted a 3-year longitudinal study with 200 Portuguese-speaking children in the last year of preschool, 1st and 2nd grades. This study analyzed cases of children identified at the 2nd grade as having dyslexia. Letter knowledge and semantic fluency were found to be the factors with the greatest predictive power of dyslexia. Considering the scarce studies in Portuguese it was the authors’ intention, with this study, to investigate a model that assesses not only cognitive and reading-related variables but also sociodemographic variables. We intend to understand if all variables’ clusters are inter associated and which are more related to reading acquisition. Finally, we intend to study children from the very beginning of first grade, so we can trace an early path to successful reading acquisition.

As a result of the inconsistencies between graphemes and phonemes, children learning in different orthographies can have more or less difficulties. For example, English-speaking children need a longer learning period (two times longer) to acquire the reading foundations (Seymour et al., 2003). Difficulties also arise for most individuals learning in non-opaque orthographies and these difficulties sustain into adolescence even in transparent orthographies (Eklund et al., 2018). European Portuguese orthography presents fewer inconsistencies than English between graphemes and phonemes – 35 phonemes for 67 graphemes (Seymour et al., 2003; Sucena et al., 2009). Among European orthographies, Portuguese is in an intermediate position, with a tendency for the opaque pole. For this reason, Portuguese children acquire reading foundations at a slower pace than children who learn to read in more transparent spellings such as Castilian or Finnish. Indeed, at the end of the 1^st^ grade, Portuguese children have an average performance of around 75% in word and pseudoword reading, in contrast with ceiling levels obtained by children who acquire reading in more transparent orthographies (e.g., Spanish children reveal an accuracy of 90% for decoding) (Seymour et al., 2003). Even if Portuguese-speaking children acquire reading skills earlier and faster than English speakers, some Portuguese children still face difficulties and struggle to develop reading and spelling skills. More studies that analyze the predictive power of these skills with children learning to read in an intermediate orthography such as European Portuguese are necessary.

In the Portuguese educational system, children initiate reading acquisition in first grade, at the age of 6. First grade is mostly focused on the Portuguese discipline, specifically on reading acquisition. The Portuguese Ministry of Education defines the essential learning outputs by grade and by discipline (Direção Geral da Educação [DGE], 2018). Regarding the 1st grade, in the Portuguese class, comprehension and expression of written language in reading and writing are included in the essential learning outcomes. Regarding preschool, its attendance is facultative. The Ministry of Education defines curricular orientations, with the purpose of training children with pre-reading abilities, such as phonemic awareness (Rodrigues et al., 2017; Direção Geral da Educação [DGE], 2018). Almost all Portuguese children (95%) attend preschool before entering 1st grade. Due to the scarcity of research on the impact of reading, cognitive, and sociodemographic-related variables in predicting reading difficulties in an intermediate orthography such as European Portuguese, this study explores the predictive power for reading success (as assessed by the final classification in Portuguese discipline) of (i) sociodemographic related variables: mothers’ education, and SES, (ii) cognitive related variables: phonological working memory and vocabulary, and (iii) reading-related variables: letter-sound knowledge (assessed through letter reading and letter spelling), phonemic awareness, and rapid naming (RAN). Taking previous information into account, it was hypothesized: all variables’ clusters would be positively and statistically significantly associated with the classification in the Portuguese discipline at first grade (H1); sociodemographic, cognitive, and reading-related variables would predict the classification in the Portuguese discipline (H2). Based on the expected results, the conduction of an analysis regarding whether letter spelling at the beginning of the first grade would mediate the relationship between mother education and the classification in the Portuguese discipline (H3).

## 2. Method

### 2.1. Participants

A total of 119 Portuguese first graders, 60 girls (50.4%) and 59 boys (49.6%), aged between 5 years old and 7 months and 6 years and 11 months (*M* = 74.4, *SD* = 3.5), from schools in the northwest of Portugal from three different SES were assessed at the beginning of school year. The SES were evaluated regarding the type of school context – public (average and low SES) or private (high SES). Regarding public schools, those integrated into “Priority Intervention Educational Territories”^1^ were classified as low SES whereas the remaining were classified as average SES. From the 119 participants, 34 children (28.6%) come from public and TEIP (Educational Territory of Priority Intervention) schools and were considered from low SES, 62 children (52.1%) come from public non-TEIP schools and were considered from medium SES, and 23 children (19.3%) attended a private school and were considered from high SES (Table 1). After analyzing the data obtained from Raven’s Matrices, one child was removed from the sample for presenting an intellectual level below the average parameters for her chronological age (Raven et al., 2009). That same child was diagnosed with Pervasive Development Disorder by the School’s Psychology team.

**Table 1:** Description of the participants (sex and age) regarding SES.

SES	*N* Total (%)	*n* Boys	*n* Girls	Age M (SD)	Raven M (SD)
Low	34	20	22	75.3 (3.4)	18.26 (4.89)
Medium	62	19	17	73.8 (3.2)	19.18 (3.84)
High	23	20	21	73.4 (3.5)	19.09 (5.01)
Total	119	59	60	74.4 (3.5)	18.73 (4.67)

### 2.2. Instruments

Parents and teachers answered sociodemographic questions regarding their children (e.g., sex, age, type of school – NTEIP/TEIP; public/private – and mother’s education).

Children were assessed with the European Portuguese Reading Assessment Battery (ALEPE, Sucena and Castro, 2011), specifically regarding letter-sound knowledge, phonemic awareness, and RAN; the WISC III (Wechsler, 2006) was adopted to assess vocabulary and phonological working memory; the Raven’s Colored Progressive Matrices (MPCR, Raven et al., 2009) was administered as a nonverbal intelligence measure.

To assess the Letter-Sound knowledge two subtests from ALEPE were adopted: letter reading and letter spelling. In the Letter Reading subtest, the child is asked to read one letter (at a time) displayed on a computer screen. This subtest consists of two training items and twenty-three experimental items. Both the sound and the letter name are accepted as correct answers. The result is the total number of letters correctly read. In the letter-spelling subtest, the child is asked to spell each letter dictated by the examiner. This subtest consists of two training items and twenty-three experimental items. The result is the total number of letters correctly written by the child (Sucena and Castro, 2011).

To assess phonemic awareness, the Phonemic Metalinguistic Awareness subtest (ALEPE) was used. This subtest evaluates the child’s ability to identify the common phoneme in a pair of words. This subtest consists of three training items and twelve experimental items. For each item, there is a pair of two-syllable words with simple and complex syllabic structures {CV [Consonant-Vowel (open syllable)] and CVC [Consonant- Vowel-Consonant (closed syllable)]}. The result is the total of correct answers (Sucena and Castro, 2011).

To assess RAN, the RAN subtest from ALEPE was adopted. The child is asked to name the visual stimuli (colors) displayed on the screen computer’ as quickly as possible. The stimuli are displayed in continuous format (4×4 colors). Before the application of the task, a training trial is conducted. The result corresponds to the total of colors accurately named (Sucena and Castro, 2011).

To assess vocabulary and phonological working memory two WISC III (Wechsler, 2006) subtests were adopted. The Vocabulary subtest consists of 30 words that the child is asked to define (orally). After four consecutive errors, the task ends (Wechsler, 2006). In the Digit Span subtest (to assess phonological working memory), the examiner dictates a sequence of digits (from 2 to 8 digits) and asks the child to repeat the sequence in the same order (series of digits in the forward direction) or reverse order (series of digits in the reverse order) (Wechsler, 2006).

To assess nonverbal intelligence, Raven’s Colored Progressive Matrices (MPCR, Raven et al., 2009) were used to screen children with cognitive disabilities. Each item corresponds to a sequence of figures with a logical pattern. The child is asked to select, among the options, the image that matches the pattern.

To assess reading competence, the classification in the Portuguese discipline was adopted. This classification has four levels – insufficient, sufficient, good, and very good. Regarding the Portuguese Educational Board of the Ministry of Education Direção Geral da Educação [DGE] (2018), the classification in the Portuguese discipline should have into consideration, among other aspects (such as orality, literary education, and grammar), the ability to decode isolated words with the correct articulation and proper prosody. At first grade, this classification considers mainly the decoding ability (Direção Geral da Educação [DGE], 2018).

### 2.3. Procedures of data collection

All recommended ethical care guidelines for this type of study were observed. The study was submitted for appreciation by an Ethics Committee (reference number 605/07–2019) and authorizations were collected from (i) Education Ministry, (ii) school boards, and (iii) those formally responsible for the child’s education (e.g., parents or other relatives), ensuring the voluntary participation of all children.

The data collection took place in two moments at the beginning of the first school term (September) and at the end of the school term (June). At the first moment (M1) participants were administered sociodemographic measures (mother’s education and SES), cognitive measures (vocabulary and phonological working memory), and reading measures (phonemic metalinguistic awareness, letter reading, letter spelling, and RAN). Raven was also administered at M1 for screening purposes.

At the second moment (M2) the final classification in Portuguese was collected. Portuguese classifications are categorized as Insufficient, Sufficient, Good, and Very Good. To run the logistic regression analysis, Portuguese classifications were computed into lower (insufficient and sufficient) and higher (good and very good). The results of the Portuguese classifications are presented in complete scores except when mentioned or when the authors are reporting the model test.

### 2.4. Procedure of data analyses

Statistical analyses were performed through the Statistical Package for the Social Sciences (SPSS IBM) for Windows, version 28.0. Statistical analyses were used to characterize the participants according to sociodemographic aspects. To analyze the correlations between all the variables assessed, parametric and non-parametric correlation coefficients tests (Person and Spearman) were conducted. Whenever the results of the non-parametric tests go in the same direction as the parametric tests, the results of the parametric test are reported (Martins, 2011).

Whenever strong correlations were found, Multiple Logistic Regression analyses were run to analyze the predictive power in the final classification in the Portuguese discipline. In this way, the regression analysis was conducted testing the predictive impact of mother education and letter spelling in the Portuguese classifications, as these were the variables that correlated stronger with the Portuguese classification. The classification in the Portuguese discipline was coded as a dichotomous dependent variable (lower Portuguese classifications/ highest Portuguese classifications) in order to allow the conduction of the Multiple Logistic Regression analysis. The groups were chosen based on a continuum between insufficient and very good classifications. Classifications between 1 and 2 (insufficient and sufficient) in one group and 3 and 4 (good and very good) in another one. The following assumptions were verified before conducting the regression analysis. The sample size was greater than 15 participants per predictor (*n* = 119), absence of singularity, absence of multicollinearity, and absence of outliers were guaranteed (Hilbe, 2017). Once the assumptions were fulfilled, the Multiple Logistic Regression analysis was pursued.

Based on the literature review and the correlations and the regression results (presented below, in the Results Section), the authors decided to test a model in which letter spelling is a mediator of the relationship between the mother’s education level and the Portuguese classification. The model was tested with an Analysis of Moment Structures (AMOS), version 28.0 for Windows. First, the following assumptions were verified: minimum size of the sample (between 100 and 200 participants) (Schumacker and Lomax, 2004); multicollinearity (*r* > 0.90, Tabachnick and Fidell, 2013), linearity (correlations statistically significant between variables, (Marôco, 2010)), normality multivariate (Mardia coefficient ≦ 3) and the lack of outliers (Mahalanobis Distance <0.001) (e.g., Garson, 2012; Tabachnick and Fidell, 2013). The minimum sample size was fulfilled as the sample has 119 participants; the multicollinearity assumption was fulfilled since variables that are linear combinations of others already integrated into the analysis were not included, as well as the relationship between predictors is shown by Tolerance = 0.99 and VIF = 1.00; the normality multivariate is not fulfilled; the outliers assumption is fulfilled once Standardized Residuals present a minimum of −1.95 and a maximum of 1.21, as well as the maximum of Cooks’ D = 0.18.

## 3. Results

### 3.1. Descriptive results

The distribution of the participants across the results in the Portuguese classification (complete scores or dichotomic score) may be observed in Table 2. Participants with classifications ranging between insufficient and good (inclusive) represent around 50% of the sample, with the other 50% representing very good classification.

**Table 2:** Description of the participants (sex and age) regarding Portuguese classifications.

	*N* Total (%)	*n* Boys	*n* Girls	Age M (SD)
PT Classifications				
Insufficient	17 (14.3%)	10	7	76.25 (4.33)
Sufficient	12 (10.1%)	2	10	76.00 (4.30)
Good	31 (26.0%)	13	18	74.35 (2.96)
Very Good	59 (49.6%)	34	25	73.74 (3.49)
PT Class. Dichotomic				
Low	29 (24.4%)	12	17	76.15 (4.1)
High	90 (75.6%)	47	43	73.97 (3.3)

Age presented in months.

The descriptions of sociodemographic, cognitive, and reading-related variables may be observed in Table 3. Regarding the sociodemographic variables, results indicate that the majority of the participants’ mothers have higher education (57%) and come from medium SES (52.1%). Regarding the cognitive variables results fall within the reference values (expected for the chronological age) both on the vocabulary test (*M* = 10.22; *SD* = 4.73) and on the digit span subtest (*M* = 7.99; *SD* = 2.24). Regarding the results of the reading-related variables, children know around 10 letter-sound relationships. Children present a mean of 6 correct answers for the Phonemic task (*M* = 6.39; *SD* = 4.35) and name 22 colors in the RAN subtest (*M* = 21.77; *SD* = 5.94). These values fall within the reference values.

**Table 3:** Descriptive results of sociodemographic, cognitive, and reading-related variables.

	Variables		*N*	%	Min-Max	Mean (SD)
Sociodemographic	1.Mother Schooling	Till 9th Grade	28	23.5%	–	–
		High School	23	19.3%	–	–
		Higher Education	68	57.1%	–	–
		TOTAL	119	100%	–	–
	2.SES	Low	34	28.6%	–	–
		Median	62	52.1%	–	–
		High	23	19.3%	–	–
		TOTAL	119	100%	–	–
Cognitive	3.Vocabulary		74	100%	0–22	10.22 (4.73)
	4.Working memory		74	100%	2–14	7.99 (2.24)
Reading	5.Letter reading		119	100%	0–23	9.16 (6.24)
	6.Letter spelling		119	100%	0–23	10.60 (6.29)
	7.PMA		119	100%	0–12	6.39 (4.35)
	8.RAN		119	100%	1–36	21.77 (5.94)

SES, Socioeconomic Status; PMA, Phonemic Metalinguistic Awareness; RAN, Rapid Automatized Naming.

The results of sociodemographic, cognitive, and reading-related variables regarding Portuguese classifications are described in Table 4. Among the children whose mothers education is at the 9th grade or below, the distribution of the Portuguese classification is uniform (*ca.* 25% for each classification). Among the children whose mothers have high school education 65% present very good scores in Portuguese. Regarding the children whose mothers have higher education, the cumulative percentage of good and very good classifications is 84%.

**Table 4:** Description of sociodemographic, cognitive, and reading-related variables by PT Classification.

Portuguese classification						
Variables			Insufficient *N*	Sufficient *N*	Good *N*	Very Good *N*
Sociodemographic	1. Mother Schooling (*n* = 119)	≤ 9th Grade	9	5	9	5
		High School	1	3	4	15
		Higher Education	7	4	18	39
	2. SES (*n* = 119)	Low	6	3	13	12
		Median	10	8	10	34
		High	1	1	8	13
			Insufficient M (SD)	Sufficient M (SD)	Good M (SD)	Very Good M (SD)
Cognitive	3. Vocabulary (*n* = 74)		7.00 (4.47)	6.60 (3.65)	10.96 (5.10)	10.92 (4.30)
	4. Working Memory (*n* = 74)		5.50 (2.27)	7.00 (2.55)	8.35 (2.57)	8.42 (1.60)
Reading	5. Letter Reading (*n* = 119)		2.65 (1.84)	5.75 (1.84)	3.71 (0.67)	6.31 (0.82)
	6. Letter Spelling (*n* = 119)		2.76 (1.39)	6.50 (2.28)	9.00 (3.88)	14.53 (5.72)
	7. PMA (*n* = 119)		3.88 (3.67)	4.42 (3.68)	6.13 (3.85)	7.64 (4.50)
	8. RAN (*n* = 119)		18.71 (7.70)	18.08 (3.48)	21.42 (6.13)	23.59 (4.98)

SES, Socioeconomic Status; PMA, Phonemic Metalinguistic Awareness; RAN, Rapid Automatized Naming.

Regarding SES, 18% of the children from low SES have insufficient results in the classification in Portuguese. Similar results were found with children from medium SES (ca 16% with insufficient results). On the other hand, only 4% of the children from high SES have insufficient results. Very good results are higher in medium and high SES (respectively 55 and 57% against 35% in low SES).

Also, results are higher for both cognitive and reading-related variables for children with better (good and very good) classifications in Portuguese. Generically, reading-related variables have a linear growth from insufficient to very good classifications in Portuguese. The letter reading results spans three letters, varying from insufficient classification in Portuguese (*M* = 2.65) to very good classification in Portuguese (*M* = 6.31). Regarding letter spelling (with the same answer scale of 23 letters as the maximum) there is a range of 12 letters between insufficient (*M* = 2.76) and very good classifications (*M* = 14.53). Even if the insufficient scores in both variables are similar (*ca.* 3 letters) the scores for children who obtain very good in Portuguese classifications are discrepant (respectively, letter reading *M* = 6.31; letter spelling *M* = 14.53). Letter spelling is the variable that stands out, with results growing around four points from insufficient scores to sufficient, two additional points from sufficient to good, and the highest slope from good to very good (around 5 letters). The variation from the insufficient and sufficient scores regarding Phonemic Metalinguistic Awareness and Rapid Automatized Naming is minor (PMA *M* = 3.88 to M = 4.42; RAN *M* = 18.71 to *M* = 18.08). A higher variation is found from sufficient to good, and from good to very good scores. This variation is around two points for PMA and around three points for RAN.

### 3.2. Correlation results

Table 5 shows the correlation analysis between all the variables assessed in M1 and between the variables assessed in M1 and the Portuguese classification in M2. All correlations between the tasks assessed in M1 and the Portuguese classifications were positive and significant, except for the SES. More specifically, positive correlations were found between the classifications in Portuguese and mother’s education (*r* = 0.33, *p* < 0.001 – children whose mothers have higher schooling tend to have better classifications in Portuguese), vocabulary (*r* = 0.28, *p* = 0.01 – children with higher scores in vocabulary tend to have higher classifications in Portuguese), phonological working memory (*r* = 0.40, *p* < 0.001 – children with higher scores in phonological working memory tend to have higher classifications in Portuguese), letter reading (*r* = 0.60, *p* < 0.001 – children with higher scores in letter reading present higher classifications in Portuguese), letter spelling (*r* = 0.68, *p* < 0.001 – children with higher scores in letter spelling also tend to have higher classifications in Portuguese), PMA (*r* = 0.33, *p* < 0.001 – higher scores in PMA tend to be associated with higher classifications in Portuguese), and RAN (*r* = 0.34, *p* < 0.01 – higher scores in RAN present higher classifications in Portuguese).

**Table 5:** Correlations between the variable clusters (sociodemographic, cognitive, reading-related) and Portuguese classification.

			*N*	1	2	3	4	5	6	7	8	9
M1	Sociodemographic measures	1. Mother schooling	119	–								
		2. SES	119	0.363***	–							
	Cognitive measures	3. Vocabulary	119	0.003	−0.045	–						
		4. Working memory	119	0.144	0.350**	0.393***	–					
	Reading measures	5. Letter reading	119	0.332***	0.278**	0.397***	0.458***	–				
		6. Letter spelling	119	0.633***	0.345***	0.343**	0.410***	0.910***	–			
		7. PMA	119	0.236**	0.074	0.343**	.205 t	0.494***	0.500***	–		
		8. RAN	119	0.266**	0.249*	0.188	0.420***	0.426***	0.437***	0.396***	–	
M2		9. Port. Classif.	119	0.325***	0.162†	0.283**	0.379***	0.596***	0.683***	0.330***	0.338***	-

***p < 0.001; **p < 0.01; *p < 0.05; †*p* < 0.10; SES, Socioeconomic Status; PMA, Phonemic Metalinguistic Awareness; RAN, Rapid Automatized Naming; Port. Classif., Portuguese Classification.

### 3.3. Model test

The logistic regression model is statistically significant, χ2 (2) = 62.75, *p* < 0.001, correctly classifying 87% of cases. Letter spelling is the only variable that proves to be a statistically significant predictor of Portuguese classifications, *B* = 0.600, Wald = 20.36, *p* = 0.001 (Table 6). The increase of 1 point in the letter spelling task is associated with an increase of 82% in the odds of receiving a higher classification in Portuguese.

**Table 6:** Summary of logistic regression analysis for variables predicting Portuguese classifications.

		Model	*B*	Wald
Reading measures	Letter spelling	χ^2^ (2) = 62.75***	0.600	20.36***
Sociodemographic measures	Mother schooling		0.217	0.364

***p < 0.001; *p < 0.05.

**Figure 1:**
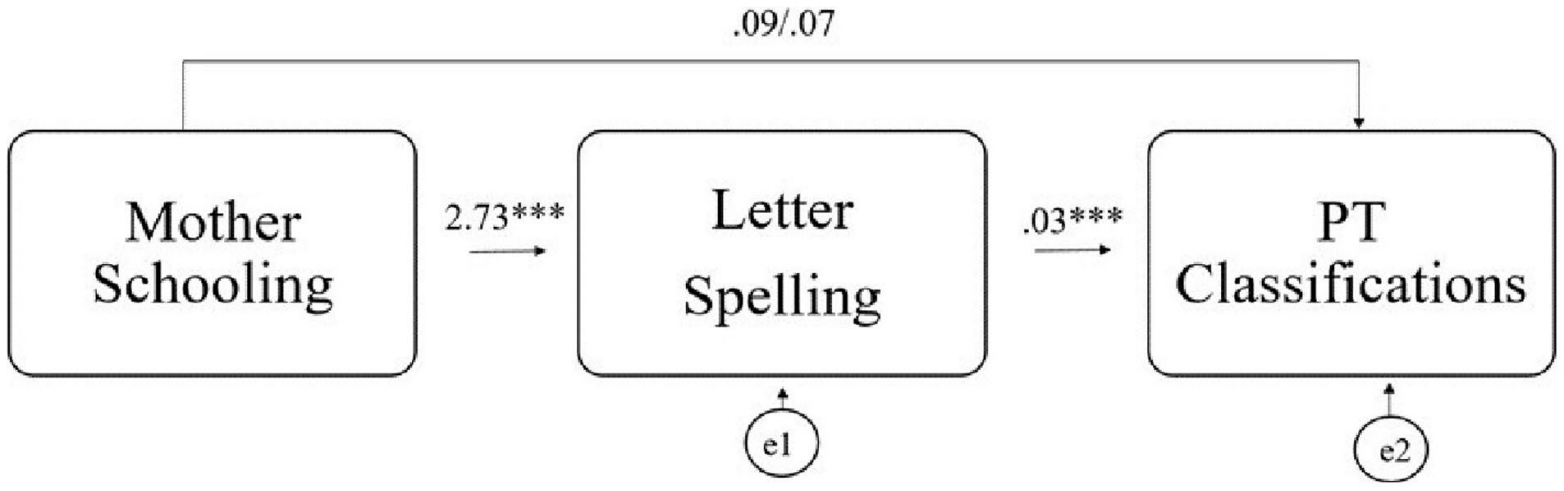
Diagram with the relationship between the variables presented in the conceptual model without outliers.

The path analysis was run to test the proposed model (letter spelling as a mediator of the relationship between the mother’s education and the Portuguese classification). The first exploratory analysis of the modification indexes results suggests excluding 3 cases. The fit indices for the model with and without these 3 cases are shown in Table 7. The model without the 3 cases revealed better adjustment to the data (RMSEA = 0.28; GFI = 0.77; AGFI = 0.74) than the group with the 3 cases (RMSEA = 0.36; GFI = 0.00; AGFI = 0.54). The RMSEA (0.28) presents a value higher than acceptable for this index (< 0.08). However, the AGFI presents a value of 0.74, slightly below the recommended value (˃0.90). According to Marôco (2010), the RMSEA indexes are classified as unacceptable (> 0.10) regardless of the group; the GFI indexes are considered bad for both groups (<0.80), as well as the AGFI indexes in both groups (< 0.80), but with better results (closer to the reference value<0.80) in the group without the 3 cases. The variation between the fit indices with and without the 3 cases may suggest a bias in these observations, which is why results without the cases were favored (Figure 1). Due to this, results without the 3 cases are preferable but not acceptable. It is important to note that the AMOS software did not suggest any more modification rates that would significantly alter these results after the elimination of the 3 cases.

**Table 7:** Adjustment indexes per model.

	RMSEA	GFI	AGFI
Model without modifications	0.36	0.00	0.54
Model with modifications	0.28	0.77	0.74

The unstandardized estimates of the model without the cases are shown in Figure 1. The results reveal statistically significant relationships between the mother’s education and letter spelling (= 2.73, *p* < 0.001), as well as between letter spelling and the Portuguese classifications (= 0.03, *p* < 0.001), and between mother’s education and the Portuguese classifications (direct unstandardized = 0.09, *p* = 0.04) but not indirectly (indirect unstandardized =0.07). The model suggests that letter spelling does not significantly mediate the relationship between the mother’s education and the classification in Portuguese discipline.

## 4. Discussion and conclusion

The identification of early predictors of reading acquisition is essential in any policy or program that aims to promote reading and spelling acquisition. Concerning opaque (e.g., English) and transparent (e.g., Finnish) orthographies, several studies have led to an installed knowledge about which associations we should pay attention to upon entering the first grade. The panorama for European Portuguese orthography is disparate as it is an intermediate orthography, for which the literature is scarce (Vaessen et al., 2010; Ziegler et al., 2010; Carvalho et al., 2017). In this way, there is no possibility of generalization based on existing studies for opaque or transparent orthographies.

This study aimed to contribute to filling this gap and to investigate whether sociodemographic variables, cognitive variables, and reading-related variables, were associated with reading acquisition in an intermediate orthography such as European Portuguese. For each variable cluster, the most robust variables (based on opaque and transparent orthographies) were selected. Regarding sociodemographic variables two possible predictors were selected: mothers’ schooling and SES. Regarding cognitive variables two variables were analyzed: vocabulary and phonological working memory. Finally, regarding reading-related variables, three variables were selected: letter-sound knowledge (letter reading and letter spelling), phonemic metalinguistic awareness, and RAN (colors). Reading acquisition was assessed based on the classification in the Portuguese discipline.

From the descriptive results, it is interesting to highlight that the students with the worst classifications in Portuguese are the older ones. These results may be supported by the literature that has stated that grade retention can have a negative effect in the areas of growth, learning, social adjustment, and classroom behavior (Griffith et al., 2010). The expectation is if students are not reaching grade level results giving an extra year in the same grade, will help them to develop the academic skills they were initially unable to demonstrate (Dombek and Connor, 2012). However, the literature has been proving that grade retention has a negative cost–benefit ratio, with less social and monetary capital than the costs to implement (Reutzel et al., 2005; Reynolds et al., 2010). When a child has learning difficulties and repeats the same grade, in the next year the difficulties tend to persist (Jimerson and Ferguson, 2007). Thus, when the learning difficulties persist, it might bring several problems inclusive in the classroom behavior. Even though it is important to reflect on these results and their impacts, these are exploratory results, and more studies are important to justify these results.

Before conducting the regression analysis, each variable cluster was analyzed regarding their correlation with the final classification obtained in the Portuguese discipline, as well as the correlation between clusters. Unsurprisingly there is a significant correlation between variables within each cluster. Indeed, these results are in line with previous literature which emphasizes the relationship between sociodemographic variables, such as schooling and SES (Aikens and Barbarin, 2008; Corso et al., 2016; Direção-Geral de Estatísticas da Educação e Ciência [DGEEC], 2016; Guo et al., 2018; Freitas et al., 2019), as well as between cognitive variables such as vocabulary and phonological working memory (Teng and Zhang, 2021) and between reading-related variables such as letter-sound knowledge, phonemic awareness (Sucena et al., 2021), and RAN as reading skills predictors.

In this study, sociodemographic variables correlate with reading-related variables. Specifically, mother education correlates with reading letters and spelling letters. SES correlates with reading and spelling letters, as well as with RAN. These results are aligned with the literature that states that having a mother with a reduced level of schooling and living in a disadvantaged socioeconomic background are risk factors at the onset of formal education especially regarding reading skills in semitransparent (Zalewska-Lunkiewicz et al., 2016) and transparent (Koponen et al., 2016) orthographies. More research is necessary to prove the impact of the sociodemographic predictors in reading acquisition in an intermediate orthography such as European Portuguese. However, based on our results it might be suggested that mother schooling is stronger than SES to explain reading acquisition. Another important aspect that contributes to this explanation is that mother’s education, most of the time reflects characteristics that are included to describe SES (prevalence of undereducated people in low-SES communities vs. prevalence of more highly educated people in high-SES communities).

Cognitive variables also correlate with reading-related variables. Specifically, better vocabulary correlates with a better performance in reading and spelling letters as well as in phonemic metalinguistic awareness. Across oral skills, vocabulary extension along with phonemic awareness plays a key role in protective pre-reading skills. The literature has been linking vocabulary with reading performance (e.g., Collazos-Campo et al., 2020; Cadime et al., 2021), especially in more transparent orthographies (Volkmer et al., 2019). Our results highlight the importance of improving oral skills before reading acquisition even in intermediate orthographies such as European Portuguese. Beginning readers use vocabulary knowledge to understand words that they encounter in print. When beginning readers pronounce a word, a connection between the pronunciation of a sequence of sounds to a word in their phonological lexicon is activated. If they find a match, they will keep reading. If a match is not found, once the word they are reading is not found in their phonological lexicon, comprehension is not successful. In other words, when the child reads a word for the first time if that word exists in her phonological lexicon, the reading process is easier once the child has a “confirmation” that she is reading correctly, and understands what has just been read. If the child decodes but the word is not stored in the phonological lexicon, then the understanding is not possible. Even if there is a correlation between vocabulary and reading-related variables, it is expected that during the alphabetic phase of reading acquisition (grapheme-phoneme conversion), the phase corresponding to the first grade, vocabulary will have a weaker association weight than in later phases where comprehension and fluency are expected. Therefore, it is important to focus on vocabulary development as part of reading instruction to support students’ reading acquisition and overall language development. By expanding their vocabulary, children can become more confident and proficient readers, which can have a positive impact on their academic success and future career prospects. Regarding phonological working memory, our results are aligned with the literature that reports this variable as less strong than other reading predictors (Caravolas et al., 2012).

In sum, all assessed variables – sociodemographic, cognitive, and reading-related - (except for SES), correlate with the Portuguese classification, with letter spelling standing out with the highest correlations. These results confirm previous literature that indicates sociodemographic, cognitive, and reading-related variables as predictors of reading success in different orthographies (Caravolas et al., 2013). Our results emphasize the main effect of letter spelling. Letter reading and letter spelling are two main tasks for the decoding process to be possible. Letter spelling is taught by emphasizing two processes in parallel: knowledge of letter tracing and training in handwriting. An important part of letter memorization is related to the gesture required for handwriting. This may be the reason why, while not yet fully mastering the letter-sound knowledge, children perform better in reading than in spelling. The stronger relationship between letter spelling and the reading process reflects, regarding letter-sound knowledge, a more demanding process, and as such the one that is mastered last. In sum, if the child spells the letter, he/she is also able to read it, but the reverse is not always the case. This is a potentially very useful result, as letter spelling may be adopted to determine cutoff points for the identification of at-risk children.

The results of the regression analysis, as expected based on the previous literature and on our correlation results, indicated letter spelling as the only predictor of the Portuguese classification. This result is coherent with the emphasis, in the classification in Portuguese in first grade, on the ability to decode isolated words. If the child masters letter-sound knowledge, the odds are higher for a better ability to decode isolated words and, in consequence, a better classification in the Portuguese discipline.

As letter spelling was a significant predictor, the authors tested a model in which letter spelling was a mediator of the relationship between the mother’s education (which was the variable that correlated most with letter spelling) and the classification in the Portuguese discipline. The authors expectation was that although the mother’s education correlated with the Portuguese classifications, this relationship would be mediated by letter spelling. The test of the model showed better results with the model without outliers and emphasized the relationship between the variables that have also been previously correlated in this study, i.e., mother education is associated with better letter spelling and, in turn, letter spelling is associated with higher results in the Portuguese discipline. It is important to mention that in both groups (with or without outliers), the indexes are weak, for which there is the need, in future studies, for replication with a larger sample since the literature indicates that this kind of analysis is sensitive to the size parameter (e.g., Tabachnick and Fidell, 2013). Also, the magnitude of the relationship between letter spelling and the results in the Portuguese discipline in the model without outliers is weak (0.03). In sum, these results are promising but more studies are necessary to be able to take general conclusions with intermediate orthographies such as European Portuguese.

As far as the authors’ knowledge goes this is a pioneer study exploring early predictors of (un)success in reading acquisition with Portuguese-speaking children first graders that include sociodemographic, cognitive, and reading-related variables. Even the scarce literature focusing on reading predictors in Portuguese that previously exist does not focus on the early predictors of reading acquisition, instead focusing on the reading and cognitive variables in second grade (Ziegler et al., 2010) or the precursors for reading comprehension with an adult population (Gonçalves et al., 2021). This study is also a contribution to filling this gap and to the early identification of at-risk children (in this specific case right at the beginning of 1st grade). The definition of (un)success predictors might also be useful for the application of more specific evaluation measures and the application of early intervention programs. Children with poor skills at the beginning of the year would benefit from the addition of a complementary intervention, more specific and specialized (Foorman et al., 2016). These children must be identified as early as possible, for which it is necessary to focus on the indicators that allow the (early) identification of those at risk of experiencing difficulties, even before they experience them. The promotion of skills that are related to reading can be promoted before the beginning of the 1st grade, in preschool (Foorman et al., 2016).

In future studies, it would be of interest to assess the association of other important sociodemographic variables such as the age of entrance to 1st grade and the number of years attending preschool.

It would also be of interest to carry out a replication of this study with two different groups: i) children with an intervention program in pre-reading skills in preschool; ii) children without a specific intervention program beyond the preschool curriculum. The Portuguese classification variable is a composite measure that involves several dimensions of reading skills. In future studies, it is pertinent to assess specific measures of each reading competence (e.g., words and pseudowords reading). This would allow more specific analyses to predict the success/failure of reading acquisition. It would also be important to continue this study with a follow-up and to monitor the participants’ reading development (e.g., assessing the impact of the predictors on fluency and reading comprehension). Such a study design would be relevant to observe the variation in the strength of predictors of (un)success in reading acquisition over time and monitor the participants’ reading development.

In conclusion, although reading acquisition predictors have been previously studied in opaque orthographies like English (e.g., Kuperman et al., 2016; Gordon et al., 2020), and transparent orthographies such as Spanish or Czech (Caravolas et al., 2013), research data is scarce regarding the impact of the main predictors in reading acquisition in European Portuguese at the beginning of the schooling (Vaessen et al., 2010; Ziegler et al., 2010; Carvalho et al., 2017). The present study intends to be a first step in the definition of simple and highly targeted assessment tasks to be adopted for the identification of at-risk children, using a model that assesses not only cognitive and reading-related variables but also sociodemographic variables. Early identification is the key to addressing intervention programs to those children in need as early as possible, preferably even before children experience unsuccessful paths in the process of reading acquisition.

## Data availability statement

The raw data supporting the conclusions of this article will be made available by the authors, without undue reservation.

## Ethics statement

The studies involving human participants were reviewed and approved by Ethics Committee (reference number 605/07-2019). Written informed consent to participate in this study was provided by the participants' legal guardian/next of kin.

## Author contributions

AS designed, supervised, and revised all the study. CG collected and inserted the data on SPSS. CM analyzed the data, reviewed the literature, and wrote the paper. ML supervised and revised the article critically. All authors contributed to the article and approved the submitted version.

## Funding

This work was supported by European Horizon 2020, under OPERAÇÃO NORTE-08-5266-FSE349 000095 and by national funds through FCT Fundação para a Ciência e a Technology, I.P., within the scope of the project “RISE - LA/P/0053/2020”.

## Conflict of interest

The authors declare that the research was conducted in the absence of any commercial or financial relationships that could be construed as a potential conflict of interest.

## Publisher’s note

All claims expressed in this article are solely those of the authors and do not necessarily represent those of their affiliated organizations, or those of the publisher, the editors and the reviewers. Any product that may be evaluated in this article, or claim that may be made by its manufacturer, is not guaranteed or endorsed by the publisher.

## Footnotes

^1^The NTEIP/TEIP program is a Portuguese government initiative, currently implemented in schools located in economically and socially disadvantaged territories, marked by poverty and social exclusion, where violence, indiscipline, abandonment and school failure are most evident (TEIP schools). The main goal of the program is to prevent and to reduce early school dropout and the indiscipline (Centro de Investigação e Estudos de Sociologia (CIES) and Instituto Universitário de Lisboa (ISCTE), 2011; Direção Geral Educação, 2021) and improving learning rates. This is a positive discrimination program aimed at supporting schools located in socioeconomically disadvantaged areas. The strategy of the TEIP program is based on a decentralizing model, focusing on the local, with the school as a central element in supporting the resolution of community problems (Centro de Investigação e Estudos de Sociologia (CIES) and Instituto Universitário de Lisboa (ISCTE), 2011).
